# The total joint arthroplasty care patterns in China during the COVID-19 pandemic: a multicenter cohort study

**DOI:** 10.3389/fpubh.2024.1357984

**Published:** 2024-10-15

**Authors:** Tianyi Zhang, Shan Wang, Li Yan, Huajuan Bai, Jiugong Guo, Jianchao Liu, Lihua Liu

**Affiliations:** ^1^Department of Medical Innovation and Research, Chinese People's Liberation Army (PLA) General Hospital, Beijing, China; ^2^Cadet Company One, Graduate School of Chinese People's Liberation Army (PLA) General Hospital, Beijing, China; ^3^Medical Service Teaching and Research Office, Joint Service College of National Defense University, Beijing, China; ^4^School of Humanities and Social Sciences, North China Electric Power University, Beijing, China

**Keywords:** COVID-19 pandemic, total joint arthroplasty, interrupted time series, healthcare pattern, surgery

## Abstract

**Background:**

The COVID-19 pandemic has profoundly affected the care practices of total joint arthroplasty (TJA) throughout the world. However, the impact of the pandemic on TJA care practices has not yet been studied in China.

**Methods:**

This retrospective multicenter cohort included patients aged 18 years or older who underwent TJA between January 2019 and December 2019 (prepandemic period) and January 2020 to December 2021 (pandemic period). Data were obtained from the medical records of 17 Chinese hospitals. Interrupted time series (ITS) analysis was used to estimate differences in monthly TJA volume, hospitalization proportion of TJA, preoperative characteristics, postoperative complications, 30-day readmissions, length of stay (LOS), and costs in inpatients undergoing TJA between the prepandemic and pandemic periods. Multivariate regression and propensity score matching (PSM) analyses were used to assess the impact of the COVID-19 pandemic on hospital complications, readmissions at 30 days, LOS, and costs at the patient level.

**Results:**

A total of 752,477 inpatients undergoing TJA in the prepandemic period, 1,291,248 in the pandemic period, with an average 13.1% yearly decrease in the volume of TJA during the pandemic. No significant changes were observed in the proportion of hospitalizations for TJA. ITS analyses showed increases in the proportion of comorbidities (8.5%, 95% CI: 3.4–14.0%) and the number of comorbidities (15.6%, 95% CI: 7.7–24.1%) in TJA cases during the pandemic, without increasing LOS, costs, complications, and readmission rates. Multivariate and PSM analyses showed 6% and 26% reductions in costs and readmission rates during the pandemic, respectively.

**Conclusions:**

The COVID-19 pandemic was associated with more severe preoperative conditions and decreased volume, costs, and readmission rates in patients undergoing TJA in China. These findings demonstrate that the COVID-19 pandemic did not have a dramatic impact on the TJA care pattern in China, which may have resulted from active and strict strategies in combating COVID-19 as well as a rapid response in hospital management.

## 1 Introduction

The 2019 coronavirus disease pandemic (COVID-19) has had an unprecedented impact on the global healthcare system, especially surgical care. Many countries have published recommendations on the suspension of non-urgent or elective procedures in response to the increase in COVID-19 cases (1, 2). For example, in the United States, the American College of Surgeons, the Centers for Medicare and Medicaid Services (CMS), and the American Academy of Orthopedic Surgeons advised postponing or canceling elective surgeries at the start of the COVID-19 pandemic (3, 4). Subsequently, total joint arthroplasty (TJA), including total hip arthroplasty (THA) and total knee arthroplasty (TKA), which comprise a substantial share of elective surgeries, has led to a dramatic reduction in resulted in a dramatic reduction in the number of cases, ranging from 30 to 94% (5–8).

Furthermore, it has been reported that the COVID-19 pandemic could affect the clinical outcomes and other practice patterns of TJA, although the results have been inconsistent. A study in India showed that the postoperative complication rate after TJA has increased significantly during the pandemic (9). Studies in the United States did not find an increase in the complication rate but a decrease in length of stay (LOS) (5, 10, 11).

Compared to other countries, China has not enforced policies that restrict or limit the number and arrangement of elective surgeries. Instead, the Chinese government has implemented a series of active strategies to prevent the spread of the virus and protect the wellbeing of citizens, including early detection and reporting of infected cases, contact tracing and quarantine management, strict border control, mandatory mask-wearing, temperature checks, and gathering restriction measures (12, 13). Hospitals designated fever clinics and isolation wards for suspected and confirmed COVID-19 cases. Patients were screened for symptoms, travel histories, and polymerase chain reaction (PCR) tests prior to admission (14, 15). However, surgical practice patterns during the COVID-19 pandemic in China, such as TJA, have never been evaluated. It remains to be clarified whether COVID-19 has had a substantial effect on TJA care in China. Therefore, this study aimed to compare the volume of cases, proportion of hospitalizations for TJA, characteristics, postoperative complications, readmissions at 30 days, LOS, and costs of patients undergoing TJA before and during the pandemic in China.

## 2 Methods

### 2.1 Data source and study population

A retrospective cohort study was conducted on adult patients who underwent THA and TKA from January 2019 to December 2021. The study extracted the cover page of medical records from hospital information systems (HIS) in 17 hospitals across various regions of China, including the middle, east, west, north, south, and Beijing (capital). These 17 hospitals adopted an unified HIS and utilized identical fields and data dictionary within the cover page, which included patients' demographic data, diagnosis and procedure information, clinical outcomes, and resource utilization. All inpatients who underwent elective TJA were queried according to the International Classification of Diseases, Ninth Revision, Clinical Modification, Third Volume (ICD9-CM3; THA: 81.51, TKA: 81.54). Elective cases were identified based on the indicators for elective admissions or emergency status. Patients aged < 18 years of age were excluded due to the rarity and distinct characteristics of TJA patients < 18. This study was exempt from institutional review board approval because all the data were historically de-identified. This study followed the Strengthening the Reporting of Observational Studies in Epidemiology (STROBE) reporting guidelines.

### 2.2 Study periods

To compare the TJA practice patterns before and during the pandemic, we defined two periods: a prepandemic period from January 1, 2019, to December 30, 2019, and a pandemic period from January 1, 2020, to December 30, 2021. We considered January 1, 2020, the cutoff date because the Chinese government started to implement strict measures to control and prevent the spread of COVID-19 in January 2020. We then divided the TJA patients into two groups based on their hospital admission date.

### 2.3 Variables and outcomes studied

For patient covariates, we included age, sex, and comorbidities (congestive heart failure, cardiac arrhythmias, and pulmonary circulation disorders). Furthermore, we calculated the Elixhauser score to measure the severity of comorbidities (the method for calculating Elixhauser score was presented in Supplementary Table S1) (16).

As outcomes, we assessed postoperative complications in the hospital, readmission within 30 days, LOS, and inflation-adjusted costs. Complications were determined from the diagnostic codes of the primary and secondary International Classification of Diseases, Tenth Revision (ICD10) and ICD9-CM3, according to the definition proposed by the Centers for Medicare & Medicaid Services (CMS) (17). Additionally, the monthly volume and proportion of hospitalizations associated with TJA, monthly average patient characteristics, and clinical outcomes were summarized to evaluate monthly changes before and during the pandemic.

### 2.4 Statistical analysis

The characteristics and outcomes of patients before and during the pandemic were described and compared. Continuous variables are expressed as mean (standard deviation) and compared using the *t*-test or Wilcoxon rank-sum test based on the distribution of the data. Categorical variables were expressed as counts (percentages) and compared using the chi-square test. Given the large sample size, the *P-*values for most statistical tests indicated significant differences, as well as effect size (the Cramér V for categorical variables and Cohen *d* for continuous variables), to estimate the difference between the two periods (18, 19).

We performed interrupted time series (ITS) analyses (20) to assess the association between pandemic onset and monthly TJA volumes, the proportion of hospitalizations for TJA, and average patient characteristics. Quasi-Poisson regressions were fitted to estimate the mean percentage change and 95% confidence intervals (95% CI) in monthly TJA volumes and the proportion of TJA hospitalizations between the two periods, adjusted for seasonality. Log-linear regression models were constructed to evaluate whether patient characteristics changed monthly during the pandemic.

To evaluate the relationship between the pandemic and outcomes (including postoperative complications, readmission within 30 days, LOS, and inflation-adjusted costs), we also conducted ITS analyses. Quasi-Poisson regressions were used for monthly in-hospital complications and 30-day readmissions, adjusted for seasonality and monthly patient characteristics. Log-linear regressions were fitted for the monthly average LOS and costs. Furthermore, we constructed multivariate regression models to evaluate the association between the pandemic and outcomes, adjusted for patient-level age, sex, number of comorbidities (Model 1), Elixhauser score (Model 2), and comorbidities (Model 3). Logistic regressions were used to estimate odds ratios (OR) and 95% confidence intervals (CI) of in-hospital complications readmissions within 30 days during the pandemic compared to the prepandemic period, and log-linear regressions were used to estimate the relative ratios and 95% CI. Additionally, propensity score matching (PSM) methods were used to further assess and validate the association between pandemics and outcomes (21). Logistic regression was firstly used to estimate the probability of TJA admission during the pandemic period according to patient age, sex, number of comorbidities of the patient (model 1), Elixhauser score (model 2), and comorbidities (model 3). The TJA cases before and during the pandemic were then 1:1 matched using a greedy matching algorithm. The caliper for matching we set is 0.1 times the standard deviation of the propensity score on the logarithmic scale. Logistic and log-linear regression models were created to estimate the relationships between pandemic and in-hospital complications, 30-day readmissions, LOS, and costs in the matched sample.

A 2-sided *P* < 0.05 was defined as significance. Statistical analyses were performed using R, version 4.0.2 (R Project for Statistical Computing).

## 3 Results

### 3.1 TJA volume and proportion of hospitalizations

**Figure 1:**
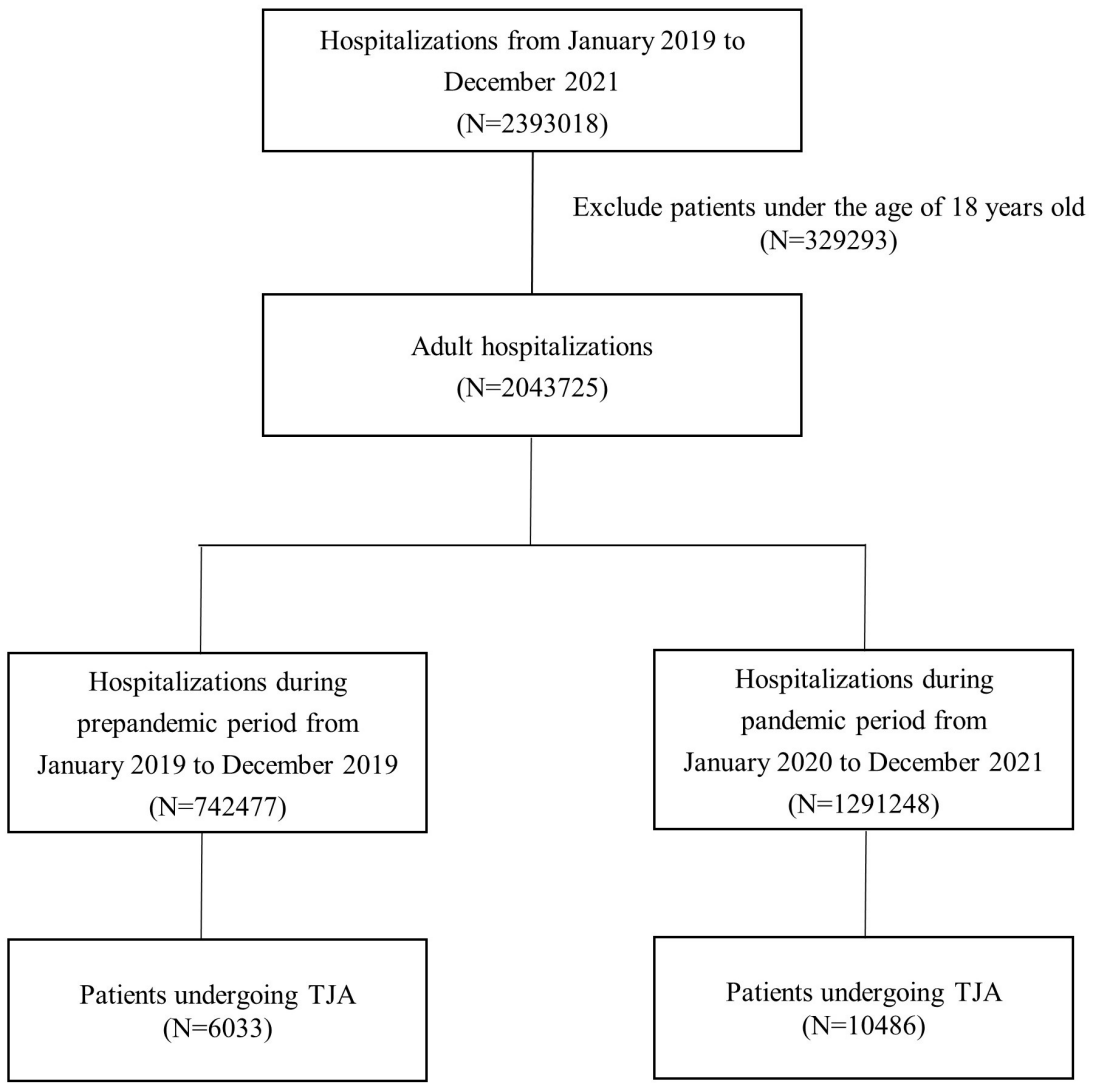
Selection flow chart for target population.

**Figure 2:**
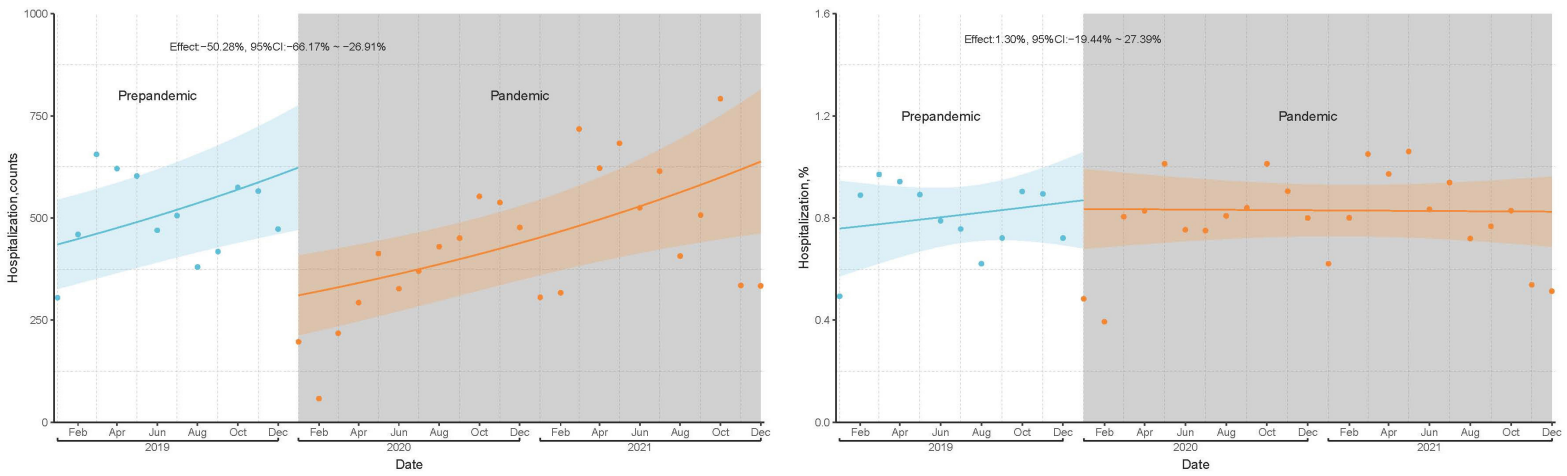
TJA volume and hospitalization proportion before and during pandemic based on ITS analyses.

In total, 2,043,725 adult hospitalizations were identified between 2019 and 2021; 752,477 were from the prepandemic period, and 1,291,248 from the pandemic period. During the prepandemic period, 6,033 of 752,477 (0.80%) patients underwent TJA, and during the pandemic period, 10,486 of 1,291,248 (0.81%) underwent TJA (Figure 1). None of the included cases were infected with COVID-19. The mean volume of TJA cases per year during the pandemic period represented a 13.1% decrease compared to 2019. At the beginning of the pandemic, there was a marked decrease in TJA cases, particularly from February to April 2020, with the monthly volume of TJA dropping by 87.4, 66.8, and 52.8%, respectively, compared to the same period in 2019. Subsequently, the monthly TJA volume gradually increased, reaching prepandemic levels. Based on the ITS analysis, there was a statistically significant difference in the average monthly volume of TJA cases before and during the pandemic period (relative difference: −50.3%; 95% CI: −66.2 to −26.9%; Figure 2A). For the monthly proportion of hospitalizations, the mean monthly proportion of TJA during the pandemic period was not significantly different from that during the prepandemic period (relative difference: 1.3%; 95% CI −19.4 to 27.4%; Figure 2B).

### 3.2 Patient characteristics

**Figure 3:**
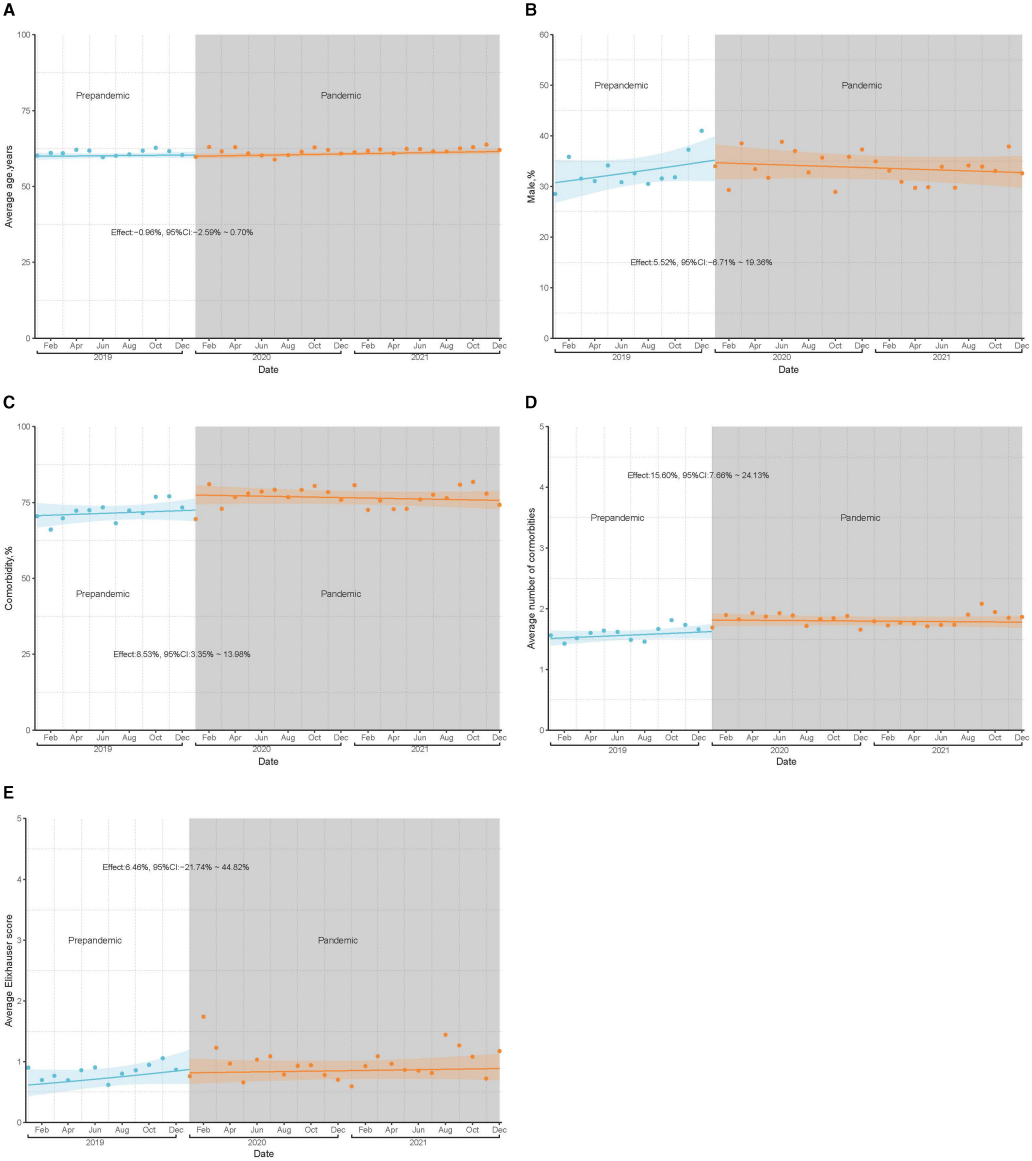
Preoperative characteristics of inpatients undergoing TJA before and during pandemic based on ITS analyses. **(A)** illustrates the average age of patients per month before and during the pandemic; **(B)** shows the proportion of male patients per month before and during the pandemic; **(C)** shows the proportion of patients with comorbidities per month before and during the pandemic; **(D)** represents the average number of comorbidities per patient per month before and during the pandemic; **(E)** shows the average comorbidity score (Elixhauser score) per patient per month before and during the pandemic.

The characteristics of the patients in the TJA group were similar before and during the pandemic, except for the number of comorbidities (Table 1). Although age, proportion of comorbidities, and Elixhauser score in patients who underwent TJA during the pandemic were significantly higher than before, absolute effect sizes were all lower than 0.1 (Table 1). Compared to the prepandemic period, patients who underwent TJA were likely to have more comorbidities (1.61 vs. 1.82), with an absolute effect size >0.1 (Table 1). ITS analyses showed that the average monthly proportion of comorbidity (relative difference: 8.5%, 95% CI: 3.4–14.0%) and many comorbidities (relative difference: 15.6%, 95% CI: 7.7–24.1%) in cases of TJA were significantly higher during the pandemic than during the prepandemic period (Figure 3).

**Table 1:** Characteristics and outcomes of patients undergoing TJA during prepandemic and pandemic period.

**Characteristics/outcomes**	**Prepandemic period (*N* = 6,033)**	**Pandemic period (*N* = 10,486)**	Effect size^*^	***P***
Age, mean (SD)	61.18 (12.70)	61.76 (12.68)	0.0457	0.0012
Male,%	2,004 (33.22)	3,487 (33.25)	0.0004	0.9617
Comorbidity,%	4,352 (72.14)	8,081 (77.06)	0.0550	< 0.0001
Number of comorbidites, mean (SD)	1.61 (1.62)	1.82 (1.75)	0.1280	< 0.0001
Elixhauser score, mean (SD)	0.83 (2.81)	0.95 (3.04)	0.0410	0.0064
**Comorbidity**				
Congestive heart failure,%	262 (4.34)	474 (4.52)	0.0041	0.5944
Caridiac arrhythmia,%	146 (2.42)	266 (2.54)	0.0036	0.6433
Valvular disease,%	15 (0.25)	25 (0.24)	0.0010	0.8976
Pulmonary circulation disorders,%	2 (0.03)	1 (0.01)	0.0084	0.6277
Peripheral vascular disorders,%	121 (2.01)	274 (2.61)	0.0191	0.0139
Uncomlicated hypertension,%	1,964 (32.55)	3,590 (34.24)	0.0171	0.0276
Comlicated hypertension,%	2 (0.03)	8 (0.08)	0.0084	0.4491
Neurological disorders,%	50 (0.83)	92 (0.88)	0.0025	0.7447
Chronic pulmonar disease,%	98 (1.62)	175 (1.67)	0.0017	0.8290
Uncomplicated diabetes,%	711 (11.79)	1,339 (12.77)	0.0144	0.0647
Complicated diabetes,%	26 (0.43)	42 (0.40)	0.0023	0.7687
Hypothyroidism,%	37 (0.61)	66 (0.63)	0.0010	0.8992
Renal failure,%	48 (0.80)	64 (0.61)	0.0109	0.1623
Liver disease,%	122 (2.02)	272 (2.59)	0.0180	0.0204
Peptic ulcer disease without bleeding,%	28 (0.46)	36 (0.34)	0.0094	0.2288
Metastatic cancer,%	6 (0.10)	6 (0.06)	0.0075	0.5027
Solid tumor without metastasis,%	7 (0.12)	29 (0.28)	0.0166	0.0331
Rheumatoid arthritis,%	281 (4.66)	462 (4.41)	0.0059	0.4521
Coagulopathy,%	17 (0.28)	34 (0.32)	0.0037	0.6358
Fluid and electrolyte disorders,%	29 (0.48)	103 (0.98)	0.0271	0.0005
Blood loss anemia,%	9 (0.15)	38 (0.36)	0.0193	0.0132
Deficiency anemia,%	2 (0.03)	11 (0.10)	0.0123	0.1952
Alcohol abuse,%	72 (1.19)	155 (1.48)	0.0118	0.1301
Depression,%	25 (0.41)	54 (0.51)	0.0070	0.3669
**Clinical outcomes**				
In-hospital complications,%	26 (0.43)	62 (0.58)	0.0096	0.2122
30-day readmissions,%	141 (2.34)	179 (1.66)	0.0237	0.0021
LOS, mean (SD), days	11.36 (10.56)	11.24 (7.23)	−0.0143	0.9767
Inflated adjusted costs, mean (SD), CNY	81,392.34 (32,147.66)	76,437.41 (31,101.05)	−0.1575	< 0.0001

^*^For categorical variables, Cramér V was used as an effect size measurement for assessing the strength of the association between two categorical variables. Cramér's V ranges from 0 to 1, indicating that it quantifies the magnitude rather than the direction of the association.

### 3.3 Clinical outcomes

Based on ITS analysis, the average monthly in-hospital complications, readmissions at 30 days, and LOS during the pandemic period did not differ significantly from those during the prepandemic period (Supplementary Figures S1–S3). There was a minor reduction in monthly average costs during the pandemic period (relative difference: −0.3%, 95% CI: −0.5 to −0.1%, Supplementary Figure S1). In the unadjusted comparisons, readmissions and costs at 30 days during the pandemic period were significantly lower than those during the prepandemic period. In multivariate regression models adjusted for the patient's age, sex, and comorbidities, there was still a 6% reduction in costs (relative ratio: 0.9, 95% CI: 0.9–1.0) and a 26% (OR: 0.7, 95% CI: 0.6–0.9) reduction in the risk of readmissions within 30 days (Table 2, Model 3). The PSM analyses showed results similar to the multivariate regression analyses (Supplementary Table S2).

**Table 2:** Association of pandemic with outcomes in patients who underwent TJA.

**Outcomes**	**Model 1** ^*^			**Model 2** ^*^			**Model 3** ^*^		
	**Effect size** ^a^	**95% CI**	***P***	**Effect size** ^a^	**95% CI**	***P***	**Effect size** ^a^	**95% CI**	***P***
Costs	0.94	0.93–0.95	*P* < 0.0001	0.94	0.93–0.96	*P* < 0.0001	0.94	0.93–0.96	*P* < 0.0001
LOS	0.99	0.98–1.00	0.1422	1.00	0.98–1.01	0.8739	1.00	0.98–1.01	0.8913
In-hospital Complications	1.04	0.65–1.67	0.8777	1.17	0.72–1.87	0.5281	1.17	0.73–1.88	0.5213
30-day readmissions	0.73	0.58–0.91	0.0049	0.73	0.58–0.91	0.0054	0.74	0.59–0.93	0.0085

^a^For costs and LOS, effect size were expressed as relative ratio estimated by log linear regressions; For in-hospital complications and 30-day readmissions, effect size were expressed as odds ratio (OR) estimated by logistic regressions.

^*^Model 1 adjusted for patient-level age, sex, and number of comorbidities; Model 2 adjusted for patient-level age, sex, and Elixhauser score; and Model 3 adjusted for patient-level age, sex, and specific comorbidities.

## 4 Discussions

Based on the available evidence, this is the first study to compare TJA practice patterns before and during the COVID-19 pandemic in China, where strict measures were implemented to prevent the spread of the COVID-19 virus at the social level, but no strategies were implemented to restrict or suspend the arrangement of elective surgeries at the hospital level. In this study, we evaluated the TJA care patterns, which account for many elective surgeries during the COVID-19 pandemic in China, evaluating the TJA volumes, the proportion of hospitalizations for TJA, patient characteristics, LOS, costs, in-hospital complications, and readmissions within 30 days.

In this study, a 13.1% annual decrease was observed in TJA volume during the pandemic period, compared directly to that in the prepandemic period. This decrease in hospitalized inpatients who underwent TJA was lower than reported in other countries, which ranged from 30 to 94% (2, 5–8, 22). Similar to other studies, there was also a steep decline in TJA volume during the early stages of the pandemic in China, which showed an 87.39% decrease in February 2020 compared to the same period in 2019. Subsequently, the volume of TJA gradually increased and recovered to prepandemic levels. In 2021, the annual volume in 2021 (6,116) was even higher than that in 2019 (6,033). The return of the TJA volume to prepandemic levels has never been reported in other studies. Heckmann et al. showed that there was also a 31.9% decrease in the peak of TJA volume in 2020 compared to before the pandemic (23). Gordon et al. observed that the TJA volume plateaued at 81.5% of the prepandemic baseline (10). Some studies attributed the decrease in TJA volume to fear of exposure to the virus in hospitals, in addition to restrictive policies (2, 24). However, the return of the TJA volume to prepandemic levels shown in our study indicated that strict strategies to prevent the spread of COVID-19 among the population could reduce the fear of contracting the virus in patients. However, the decrease in TJA volume increased to 50.28% when ITS analysis was performed. Unlike previous studies that estimated the reduction in TJA cases based on the assumption that the TJA volume would be stable if there were no pandemic (6, 23, 25), the ITS analyses used in this study evaluated the pandemic effect, controlling for secular trends in the data. The demand and volume of TJA have been reported to increase every year (26). Therefore, the actual reduction in TJA cases would be greater than estimated or reported if the increase in demand for TJA was considered.

This study did not show a change in the proportion of hospitalizations associated with TJA during the pandemic. Although there is a lack of prior research specifically addressing the proportion of hospitalizations during the pandemic, it has been reported there has been a notable shift in TJA cases from hospitalized to outpatient settings in some countries like the United States (5, 10, 24). Therefore, it is reasonable to speculate that the proportion of hospitalized TJA patients may decrease during the pandemic in these countries.

Contrary to previous studies reporting that patients undergoing TJA were younger and healthier during the pandemic period (5, 11), the results of this study indicated that patients undergoing TJA were older and had more comorbidities compared to patients with TJA before the pandemic. The number of comorbidities in patients undergoing TJA during the pandemic period was higher in both direct comparisons with the prepandemic period and the ITA analysis, indicating that Chinese hospitals tended to admit patients with severe disease status during the pandemic period.

Unlike studies conducted in the United States (5, 8, 10), our study did not show a reduction in LOS. Furthermore, the average LOS of patients who underwent TJA was much longer than that reported in the United States during and before the pandemic period. The average LOS in our study was >10 days; however, it was < 2 days in studies from the United States (5, 8, 10). This difference may be due to the accelerated shift to outpatient settings and the same-day discharge after TJA in the United States. Whether a shorter LOS could increase the rate of complication in patients undergoing TJA is inconsistent. Therefore, special caution should be exercised to shorten the LOS. Future research is required to evaluate the effect of shorter LOS on the safety of patients undergoing TJA to improve the efficiency and safety of TJA care. Additionally, our study showed a significant decrease in costs among patients undergoing TJA during the pandemic compared to those before the pandemic. The decrease in cost may be due to the Centralized Volume-Based Procurement of High-Value Medical Consumables Policy implemented in China, which aims to reduce the price and expenditure of Medical Consumables (27). The Centralized Volume-Based Procurement of High-Value Medical Consumables was first published in the middle of 2019 and carried out at the end of 2019. Therefore, the effect of this centralized volume-based procurement policy on costs may begin to manifest during the pandemic.

Several studies have assessed the impact of the COVID-19 pandemic on readmission and complication rates in patients who have undergone TJA. A study in India reported an increase in complications in patients undergoing TJA during the pandemic period (9). However, studies conducted in the United States did not produce the same results. Gordon et al. (5) and Abdelaal et al. (24) reported no changes in 30-day complications or readmissions among patients undergoing TJA. A study by Shah et al. revealed that the 30-day readmission rate was lower in the initial period of the pandemic but similar in the later period of the pandemic compared to that before the pandemic (11). However, patients who underwent TJA during the pandemic period in those studies were younger and healthier and had a lower possibility of postoperative complications. In this study, it was observed that there was no change in complication rates and a significant decrease in 30-day readmission rates in patients undergoing TJA, despite their worse conditions during the pandemic period compared to the pre-pandemic period. These findings demonstrated that the quality and safety of TJA care in China were not affected by the pandemic.

In addition to TJA, the COVID-19 pandemic has been observed to have a similar impact on other elective surgical procedures worldwide. Various specialties, including cardiac surgery, endocrine surgery, urologic oncology surgery, and neuro-oncology surgery, have also experienced a decline in surgical volume during the pandemic, particularly at its onset (28–31). A study on cervical spine surgery in the United States observed greater comorbidity burden, which aligns with the observation in this study (32). However, different from our findings, it was noticed an increase in complication rates (32). Further studies are warranted to comprehensively understand the impact of the pandemic on other elective surgical procedures in China.

This study has several limitations. First, the data used in our study were obtained from 17 hospitals and did not cover all TJA procedures performed in China. However, the 17 hospitals were geographically diverse, representing different regions of China, including the Middle, East, West, North, South, and Capital. Therefore, they were representative of Chinese hospitals. The data from these hospitals were useful in determining trends in TJA care. Second, postoperative outcome variables, including complications and readmissions, were limited to in-hospital and 30 days after the operation. Future studies should evaluate long-term follow-up for complications. Finally, this study assumed that the effects observed were directly related to COVID-19 when there were confounding factors that could have influenced clinical practice, such as a centralized volume-based procurement policy. However, we made every effort to control for observable confounders, including secular trends and patient conditions, using ITS, multivariate regression, and PSM analyses.

## 5 Conclusions

In conclusion, the results of this study revealed a different practice pattern in the care provided to inpatients with TJA in China than in other countries. The volume of TJA procedures experienced a sharp decline at the onset of the pandemic, followed by a gradual increase and eventual return to pre-pandemic levels, with no significant change in the proportion of hospitalizations associated with TJA. Furthermore, it was observed that patients who underwent TJA during the pandemic were generally older and less healthy. However, despite these challenges, there was no increase in complications, readmission rates, length of hospital stay, or costs during the pandemic period. Notably, a significant reduction in costs and readmission rates within 30 days were observed during this period. Our findings suggest that the pandemic has not had a profound negative impact on the care of TJA in China.

## Data Availability

The data analyzed in this study is subject to the following licenses/restrictions: the data presented in this study are available on request from the corresponding author. Requests to access these datasets should be directed to liulihua07@yeah.net.
