# Supplementary material for: The total joint arthroplasty care patterns in China during the COVID-19 pandemic: a multicenter cohort study
Source: Front Public Health. 2024 Oct 15;12:1357984. doi: 10.3389/fpubh.2024.1357984 (PMC11518710; doi:10.3389/fpubh.2024.1357984)
Supplement: Supplementary file 1 [file Data_Sheet_1.PDF]

Contents of Supplementary Material

Table S1 The Agency for Healthcare Research and Quality(AHRQ) Mortality Weights for Calculating Elixhauser Comorbidity

Table S2 Association of Pandemic with Outcomes in Patients who underwent TJA in PS analyses

Figure S1 Clinical Outcomes of Inpatients undergoing TJA before and during pandemic based on ITS analyses adjusted for age, sex and Elixhauser score.

Figure S2 Clinical Outcomes of Inpatients undergoing TJA before and during pandemic based on ITS analyses adjusted for age, sex and number of comorbidities.

Figure S3 Clinical Outcomes of Inpatients undergoing TJA before and during pandemic based on ITS analyses adjusted for age, sex and proportion of comorbidities.

Table S1 The Agency for Healthcare Research and Quality(AHRQ) Mortality Weights for Calculating Elixhauser Comorbidity

No.	Comorbidity	Weight
1	Congestive Heart Failure	9
2	Caridiac Arrhythmia	0
3	Valvular Disease	0
4	Pulmonary Circulation Disorders	6
5	Peripheral Vascular Disorders	3
6	Hypertension combined complicated and uncomlicated	-1
7	Paralysis	5
8	Other Neurological Disorders	5
9	Chronic Pulmonary Disease	3
10	Diabetes Uncomplicated	0
11	Diabetes Complicated	-3
12	Hypothyroidism	0
13	Renal Failure	6
14	Liver Disease	4
15	Peptic Ulcer Disease excluding bleeding	0
16	Acquired immune deficiency syndrome (AIDS)	0
17	Lymphoma	6
18	Metastatic Cancer	14
19	Solid Tumor without Metastasis	7
20	Rheumatoid Arthritis/collagen	0
21	Coagulopathy	11
22	Obesity	-5
23	Weight Loss	9
24	Fluid and Electrolyte Disorders	11
25	Blood Loss Anemia	-3
26	Deficiency Anemia	-2
27	Alcohol Abuse	-1
28	Drug Abuse	-7
29	Psychoses	-5
30	Depression	-5

Note: The Elixhauser comorbidity index scores for each case are calculated as a weighted sum of 30 comorbidities based on their presence or absence.

Table S2 Association of Pandemic with Outcomes in Patients who underwent TJA in PS analyses

Outcomes	Model1				Model2				Model3		
	Effect Size*	95%CI	P		Effect Size*	95%CI	P		Effect Size*	95%CI	P
Costs	0.94	0.93-0.96	P<0.001	.	0.94	0.93-0.96	P<0.001	.	0.94	0.93-0.96	P<0.001
LOS	0.99	0.97-1.01	0.21	.	1.00	0.98-1.02	0.92	.	1.00	0.98-1.02	0.97
In-hospital Complications	1.50	0.92-2.50	0.11	.	1.29	0.76-2.23	0.35	.	1.19	0.71-2.03	0.51
30-day readmissions	0.63	0.48-0.82	P<0.001	.	0.78	0.60-0.99	0.05	.	0.72	0.56-0.94	0.01

*For costs and LOS, effect size were expressed as relative ratio estimated by log linear regressions; For in-hospital complications and 30-day readmissions, effect size were expressed as odds ratio (OR) estimated by logistic regressions

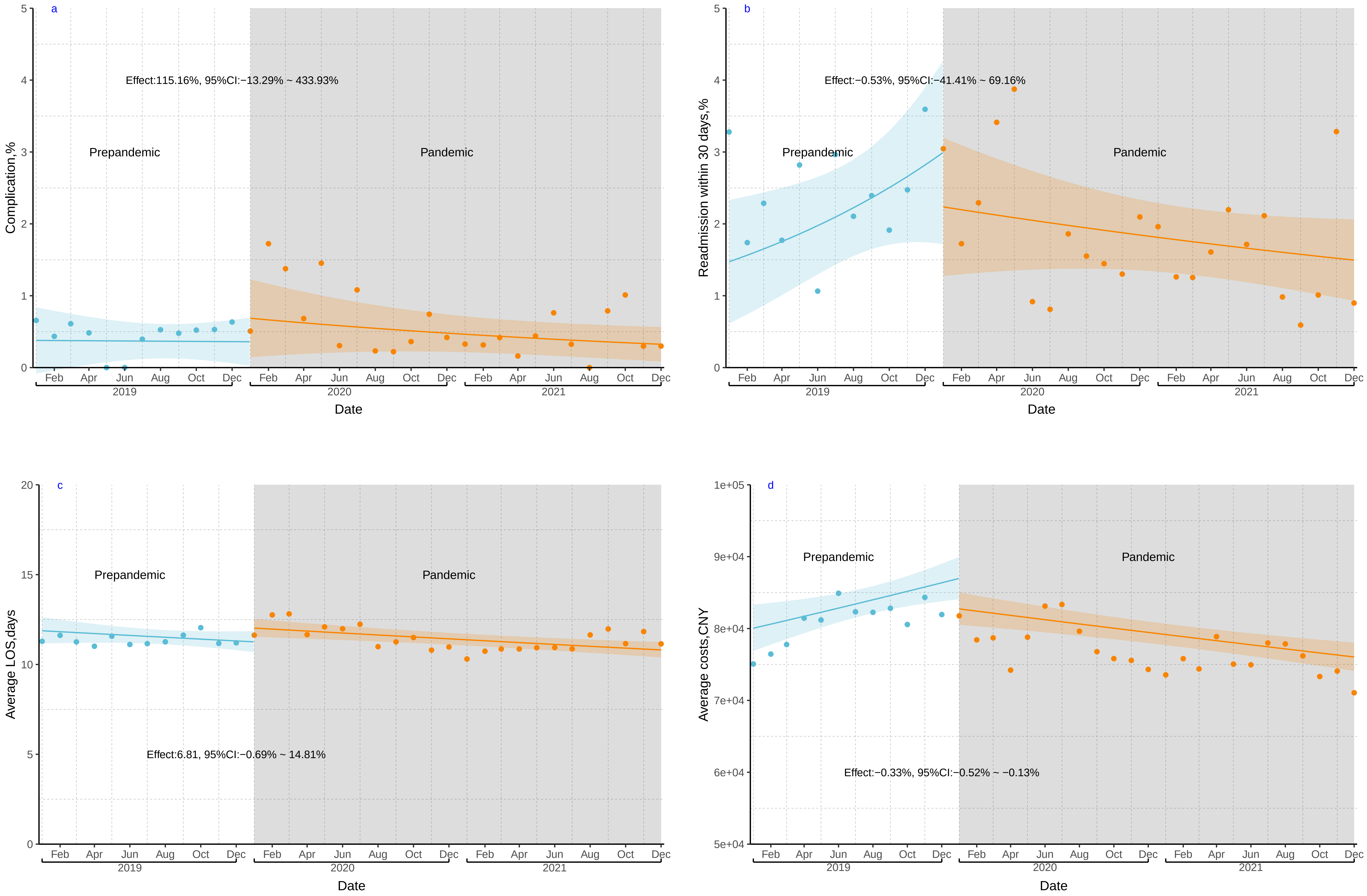

a
Effect:115.16%, 95%CI:−13.29% ~ 433.93%
Prepandemic
Pandemic
Complication,%
Feb
Apr
Jun
Aug
Oct
Dec
2019
2020
2021
Date
b
Effect:−0.53%, 95%CI:−41.41% ~ 69.16%
Prepandemic
Pandemic
Readmission within 30 days,%
Date
c
Prepandemic
Pandemic
Effect:6.81, 95%CI:−0.69% ~ 14.81%
Average LOS,days
Date
d
Prepandemic
Pandemic
Effect:−0.33%, 95%CI:−0.52% ~ −0.13%
Average costs,CNY
1e+05
9e+04
8e+04
7e+04
6e+04
5e+04
Date

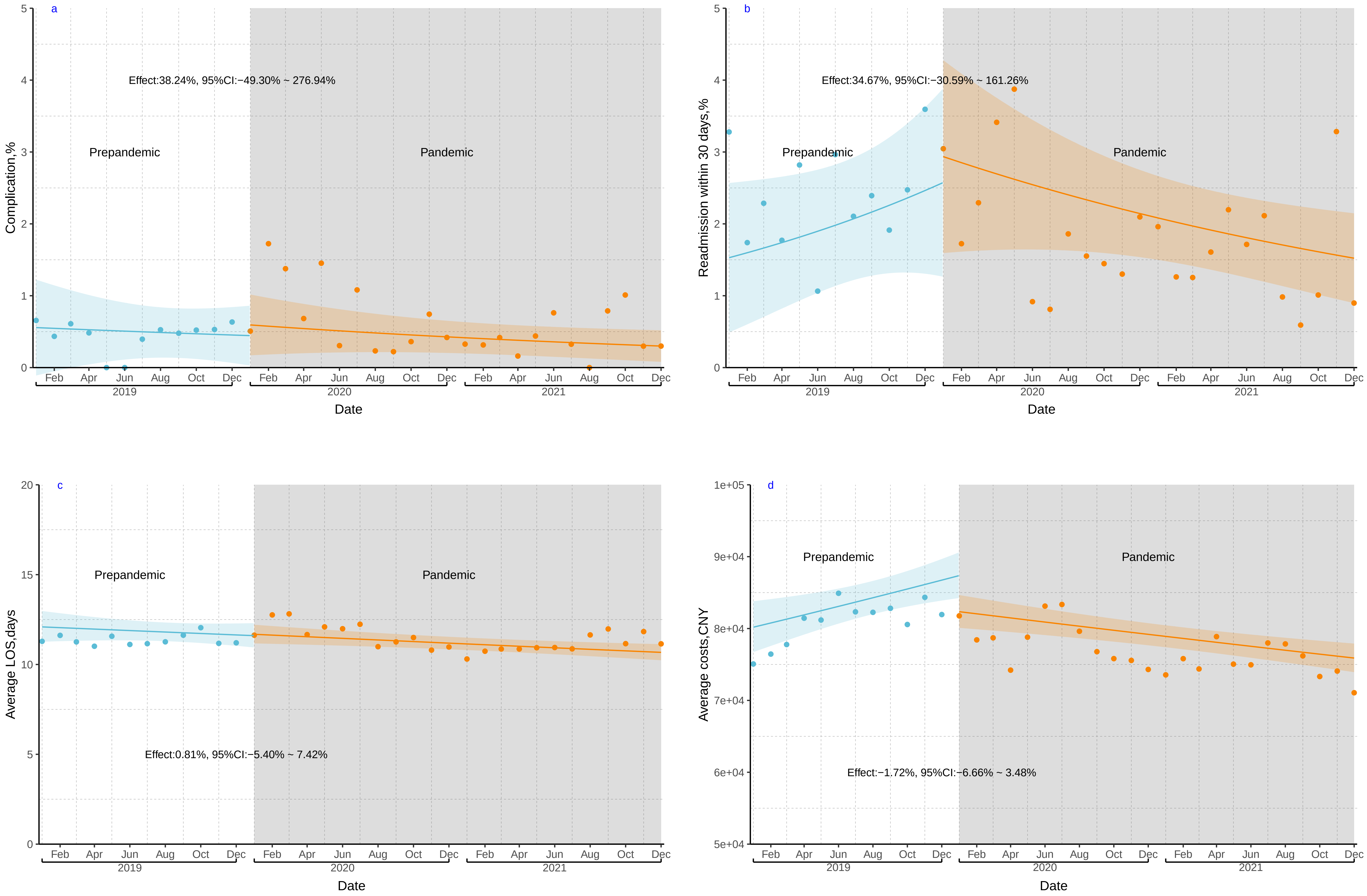
a
Complication,%
Effect:38.24%, 95%CI:−49.30% ~ 276.94%
Prepandemic
Pandemic
0
1
2
3
4
5
Feb Apr Jun Aug Oct Dec Feb Apr Jun Aug Oct Dec Feb Apr Jun Aug Oct Dec
2019
2020
2021
Date
b
Readmission within 30 days,%
Effect:34.67%, 95%CI:−30.59% ~ 161.26%
Prepandemic
Pandemic
0
1
2
3
4
5
Feb Apr Jun Aug Oct Dec Feb Apr Jun Aug Oct Dec Feb Apr Jun Aug Oct Dec
2019
2020
2021
Date
c
Average LOS,days
Prepandemic
Pandemic
Effect:0.81%, 95%CI:−5.40% ~ 7.42%
0
5
10
15
20
Feb Apr Jun Aug Oct Dec Feb Apr Jun Aug Oct Dec Feb Apr Jun Aug Oct Dec
2019
2020
2021
Date
d
Average costs,CNY
Prepandemic
Pandemic
Effect:−1.72%, 95%CI:−6.66% ~ 3.48%
5e+04
6e+04
7e+04
8e+04
9e+04
1e+05
Feb Apr Jun Aug Oct Dec Feb Apr Jun Aug Oct Dec Feb Apr Jun Aug Oct Dec
2019
2020
2021
Date

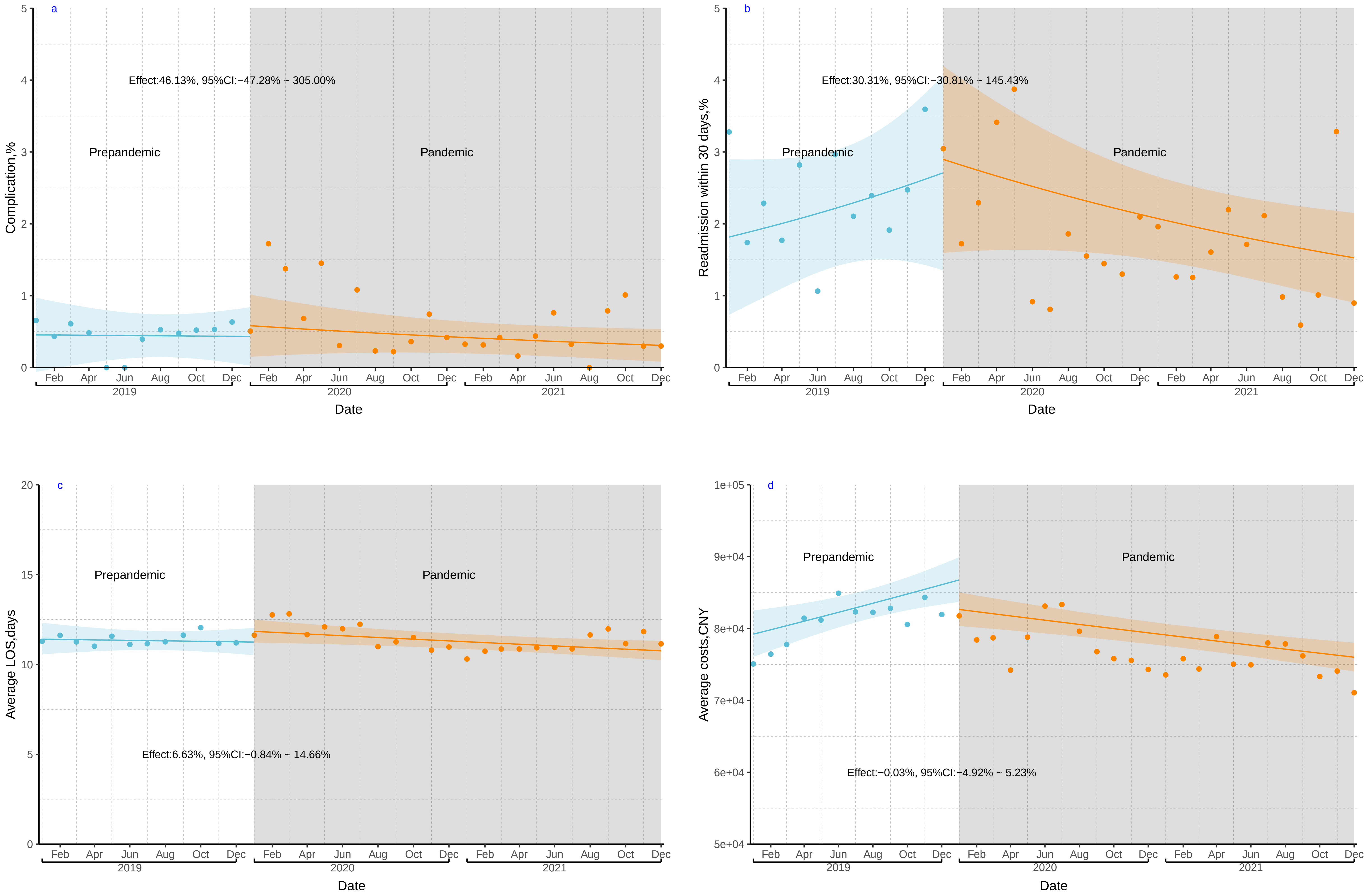
a
Effect:46.13%, 95%CI:−47.28% ~ 305.00%
Prepandemic
Pandemic
Complication,%
Date
b
Effect:30.31%, 95%CI:−30.81% ~ 145.43%
Prepandemic
Pandemic
Readmission within 30 days,%
Date
c
Prepandemic
Pandemic
Effect:6.63%, 95%CI:−0.84% ~ 14.66%
Average LOS,days
Date
d
Prepandemic
Pandemic
Effect:−0.03%, 95%CI:−4.92% ~ 5.23%
Average costs,CNY
Date
2019
2020
2021
